# The potential relationship between uric acid and the recovery in sudden sensorineural hearing loss

**DOI:** 10.1016/j.bjorl.2023.101368

**Published:** 2023-11-22

**Authors:** Yandan Zhou, Jie Wen, Zhongchun Yang, Ruifang Zeng, Wei Gong, Qiancheng Jing

**Affiliations:** a Changsha Aier Eye Hospital, Aier Eye Hospital Group, Changsha, Hunan, China; b University of South China, Hengyang Medical School, The Affiliated Changsha Central Hospital, Department of Otolaryngology Head and Neck Surgery, Changsha, Hunan, China; c University of South China, Changsha, Hengyang Medical School, Institute of Otolaryngology Head and Neck Surgery, Changsha, Hunan, China; d University of South China, Hengyang Medical School, The Affiliated Changsha Central Hospital and Institute of Otolaryngology Head and Neck Surgery, Changsha, Hunan, China

**Keywords:** Sudden sensorineural hearing loss, Uric acid, Hyperuricemia, Hearing recovery, Biomarker

## Abstract

**Objective:**

Serum uric acid is proven to be associated with chronic hearing loss, but its effect on Sudden Sensorineural Hearing Loss (SSNHL) is unclear. This study aims to evaluate the prognostic values of serum uric acid levels in SSNHL patients.

**Methods:**

The clinical records of SSNHL patients were retrospectively reviewed. Patients were divided into different groups based on hearing recovery and audiogram type, and uric acid levels were compared. Based on uric acid levels, patients were categorized into normouricemia and hyperuricemia groups, and clinical features and hearing recovery were evaluated. Univariate and multivariate analyses were performed to identify prognostic factors.

**Results:**

In total, 520 SSNHL patients were included in this study, including 226 females and 294 males. In female patients, 186 patients were included in the normouricemia group, and 40 patients were enrolled in the hyperuricemia group. Significant differences were observed in uric acid levels, Total Cholesterol (TC), rate of complete recovery, and slight recovery between the two groups. In male patients, 237 subjects were categorized into the normouricemia group, and 57 patients were included in the hyperuricemia group. The rate of complete recovery and slight recovery was lower in the hyperuricemia group compared to the normouricemia group. All patients were further divided into good recovery and poor recovery groups based on hearing outcomes. The uric acid levels, initial hearing threshold, rate of hyperuricemia, and TC were lower in the good recovery group than the poor recovery group both in female and male patients. Binary logistic regression results showed that uric acid levels, initial hearing threshold, and hyperuricemia were associated with hearing recovery.

**Conclusion:**

Hyperuricemia might be an independent risk factor for hearing recovery in SSNHL patients. Serum uric acid and initial hearing threshold possibly affected the hearing outcome in males and females with SSNHL.

**Level of evidence:**

Level 4.

## Highlights

• Hyperuricemia is an independent risk factor for hearing recovery in SSNHL.
• Serum uric acid levels negatively affect hearing outcome in SSNHL.
• Initial hearing threshold is a risk factor for SSNHL recovery.

## Introduction

Sudden Sensorineural Hearing Loss (SSNHL) is a prevalent and concerning condition characterized by a sudden onset of hearing loss of at least 30 decibels (dB) affecting three or more consecutive frequencies within 72 h or less.^1^, ^2^ Recent epidemiological investigations showed that the estimated incidence of SSNHL was at least 5–20 per 100,000 individuals, and the incidence exhibited an increasing trend.^3^ The underlying causes of SSNHL remain unclear, and several potential factors have been suggested as potential etiologies, including infections, autoimmune reactions, traumatic events, metabolic disorders, and vascular abnormalities.^4^, ^5^, ^6^, ^7^ Emerging evidence has indicated a strong link between cardiovascular risk factors and the development and prognosis of SSNHL. These risk factors include metabolic syndrome, diabetes, hypertension, and hypercholesterolemia.^1^, ^8^, ^9^ However, there is a lack of research examining the impact of serum uric acid levels on the recovery of hearing in SSNHL patients.

Serum uric acid is the end product of purine metabolism, which is under the control of the enzyme xanthine oxidase.^10^, ^11^ Accordingly, uric acid concentrations were related to age, sex, renal function, diet, and physical characteristics.^12^, ^13^ Previous studies demonstrated that abnormal uric acid exhibited an adverse effect on coagulation function, endothelial metabolic balance and inflammation response, and elevated serum uric acid levels were closely associated with increased risk of hypertension, metabolic syndrome, and cardiovascular diseases.^13^, ^14^ Experimental and epidemiological evidence suggested that uric acid and hyperuricemia acted crucial roles in cardiovascular and renal diseases and cardiovascular events.^15^, ^16^ Recently, several studies proved that uric acid metabolism was disturbed in age-related hearing impairment patients, and elevated uric acid concentrations were proven to be inversely associated with hearing thresholds for pure tone audiometry.^17^, ^18^, ^19^ Sahin et al.^20^ found that gout patients were more susceptible to suffering a high frequency of hearing loss than healthy controls. Despite these findings, few of them have focused on the implications of uric acid in SSNHL patients, and the impact of uric acid on hearing recovery was not clear. Here, we evaluate the serum uric acid levels in SSNHL patients and their prognostic values in male and female patients and investigate whether it was a risk factor in this specific population.

## Methods

### Patients and settings

A retrospective analysis was conducted on the medical records of patients diagnosed with SSNHL at our medical center from January 2018 to March 2022. Diagnosis of SSNHL was based on pure tone audiometry and adherence to established diagnostic criteria. All patients underwent comprehensive assessments including detailed medical history, physical examination, audiological tests, laboratory investigations, and magnetic resonance imaging. Exclusion criteria encompassed patients below 18 years of age, previous history of audiological or otology diseases, identifiable etiologies such as acoustic neuroma, ear surgery, ototoxicity deafness, acoustic trauma, and Meniere's disease, presence of acute inflammation, and incomplete clinical records.

We collected a range of demographic and clinical data from the patients, which included age, Body Mass Index (BMI), gender, the onset of treatment, initial hearing threshold, contralateral hearing threshold, presence of tinnitus and vertigo, serum uric acid levels, Systolic Blood Pressure (SBP), Diastolic Blood Pressure (DBP), creatinine and urea levels, Fasting Blood Glucose (FBG) and HbA1c levels, Triglyceride (TG) and Total Cholesterol (TC) levels, as well as levels of Low-Density Lipoprotein Cholesterol (LDL-C) and High-Density Lipoprotein Cholesterol (HDL-C). We also analyzed audiogram patterns and assessed the outcomes of hearing recovery. The presence of comorbidities such as diabetes mellitus, hypertension, hyperlipidemia, and hyperuricemia was determined based on the patient's medical history or newly diagnosed by a physician. This retrospective study was approved by the Human Ethical Committee of the Affiliated Changsha Central Hospital, Hengyang Medical School, University of South China (nº 2023013). Given that the study did not involve any sensitive patient information or commercial interests, it was deemed exempt from the requirement of informed consent by the Ethics Committee.

### Definitions for comorbidities and group settings

Hyperuricemia was defined as a serum uric acid level equal to or above 420 μmoL/L (7.0 mg/dL) in males and 357 μmoL/L (6.0 mg/dL) in females, in line with previous publications.^21^ The diagnosis of diabetes mellitus was based on criteria such as Fasting Blood gGlucose (FBG) levels equal to or above 7.0 mmoL/L, 2-h plasma glucose levels equal to or above 11.1 mmoL/L, or a clear medical history indicating diabetes. Hypertension was defined as repeated blood pressure measurements equal to or above 140/90 mmHg or the use of antihypertensive medications. Hyperlipidemia was diagnosed when Total Cholesterol (TC) levels were equal to or above 5.72 mmoL/L, Triglyceride (TG) levels were equal to or above 1.70 mmoL/L, or there was a documented medical history indicating hyperlipidemia.^9^, ^22^, ^23^ Based on their uric acid concentrations, both male and female patients were categorized into either the normouricemia group or the hyperuricemia group, and differences in variables and gender were compared between the two groups. All comorbidities were subject to consultation with physicians to formulate a comprehensive treatment plan. In the hyperuricemia group, all patients received diuretics, uricosuric agents, and dietary health management to control the serum uric acid level during the SSNHL treatment and follow-up period.

### Treatment protocol and hearing recovery evaluation

Before initiating treatment, all participants underwent pure tone audiometry tests to assess their hearing status. The audiogram type was classified as ascending, descending, flat, or profound based on previous studies.^24^, ^25^ In our department, SSNHL patients were treated following a standard treatment protocol, which included oral steroids and adjuvant blood-flow-promoting agents, as suggested in prior publications.^26^, ^27^ Prednisolone was administered orally at a morning dose of 1 mg/kg/day for a duration of 5 days, followed by a 5-day tapering period. Intravenous administration of 20 μg alprostadil in 100 mL of normal saline was carried out for a total of 10-days. To monitor treatment outcomes, pure tone audiometry tests were performed every 2–3 days, and the decision to terminate treatment depended on changes in audiological results. After the completion of the treatment, all patients underwent pure tone audiometry tests again. Subsequently, they underwent pure tone audiometry tests every two weeks, and were followed up for a minimum of 3-months after treatment, and the degree of hearing recovery was identified at 3-months after treatment and categorized as complete recovery, marked recovery, slight recovery, or no recovery, according to Siegel's criteria.^28^ For analysis, complete recovery and marked recovery were considered good recovery, while slight recovery and no recovery were classified as poor recovery.

### Statistical analysis

Continuous variables with a normal distribution were reported as mean ± Standard Deviation (SD), while those without a normal distribution were presented as median and Interquartile Range (IQR). Categorical variables were expressed as numbers and percentages. Statistical comparisons between groups for continuous variables were performed using either Student's *t*-test or Mann-Whitney *U* test, depending on the data distribution. The Chi-Square test was used for categorical variables. Prognostic factors in both females and males with SSNHL were identified through univariate and multivariate analyses. All statistical analyses were conducted using IBM SPSS Statistics software, version 23.0. A *p-*value of less than 0.05 was considered statistically significant.

## Results

### Demographic and clinical characteristics of patients with SSNHL

**Figure 1:**
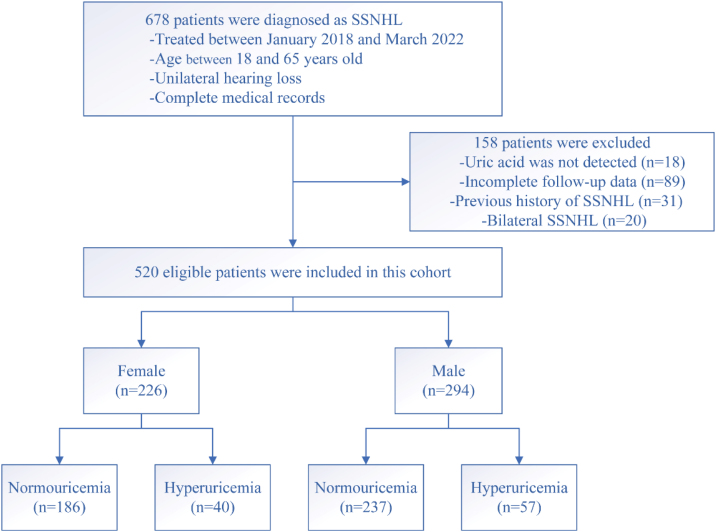
A flow chart for the study design and study population. SSNHL, Sudden Sensorineural Hearing Loss.

**Figure 2:**
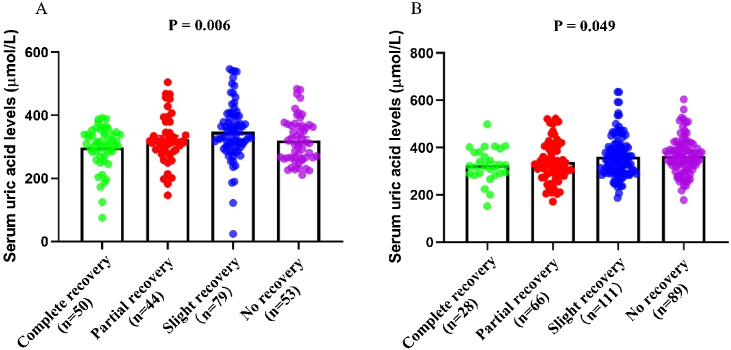
The serum uric acid levels in hearing recovery subgroups in female (A) and male (B) patients with SSNHL. SSNHL, Sudden Sensorineural Hearing Loss.

Of the total, 520 SSNHL patients (294 males and 226 females) of the original population of 678 patients met the inclusion criteria (Fig. 1). Table 1 presented the demographics and clinical characteristics of all patients, and females and males separately. The uric acid, creatinine, and urea levels were significantly higher in the male group than in the female group (*p* < 0.001). However, no statistical difference was observed in other variables between the two groups (*p* > 0.05). As Fig. 2 displayed, no statistical difference was found in uric acid levels among ascending, descending, flat, and profound groups in both female patients and male patients (*p* >  0.05).

**Table 1:** Demographics and clinical characteristics of total patients and each gender group.

Variables	Total (*n* = 520)	Female (*n* = 226)	Male (*n* = 294)	*p*-value
Uric acid, μmoL/L	333.0 (290.0, 389.2)	318.9 (282.0, 367.3)	343.6 (295.4, 401.5)	<0.001
Age, year	46 (37,52)	46 (38,53)	45 (35,51)	0.912
BMI, kg/m^2^	23.1 (21.3, 25.1)	23.4 (21.5, 25.2)	22.5 (21.2, 24.3)	0.897
Onset of treatment, day	8 (4, 13)	10 (4, 14)	7 (3, 12)	0.245
Side, left/right	265/255	123/103	142/152	0.185
Initial hearing threshold, dB	56.2 (37.8, 81.6)	55.4 (36.5, 80.9)	56.7 (37.9, 82.5)	0.219
Contralateral hearing threshold, dB	20.5 (15.6, 25.2)	21.0 (16.5, 26.1)	20.3 (15.9, 25.5)	0.589
Tinnitus, *n* (%)	335 (64.4)	149 (65.9)	185 (62.9)	0.519
Vertigo, *n* (%)	179 (34.4)	79 (35.0)	100 (34.0)	0.853
Diabetes mellitus, *n* (%)	66 (12.7)	29 (12.8)	37 (12.6)	1.000
Hypertension, *n* (%)	94 (18.1)	40 (17.7)	54 (18.4)	0.909
Hyperlipemia, *n* (%)	114 (21.9)	52 (23.0)	62 (21.1)	0.669
Hyperuricemia n (%)	97 (18.7)	40 (17.7)	57 (19.4)	0.651
Creatinine, μmoL/L	75.0 (63.8, 85.0)	63.0 (55.0, 70.0)	81.0 (72.7, 89.5)	<0.001
Urea, mmoL/L	4.8 (4.0, 5.7)	4.5 (3.8, 5.4)	4.9 (4.1, 5.9)	<0.001
SBP, mmHg	122.0 (113.0, 133.5)	122.0 (114.0, 133.0)	123.0 (112.0, 134.0)	0.869
DBP, mmHg	79.0 (72.0, 85.0)	79.0 (72.0, 85.0)	79.0 (73.0, 85.0)	0.937
FBG, mmoL/L	4.9 (4.6, 5.4)	4.9 (4.5, 5.3)	4.9 (4.6, 5.5)	0.965
HbA1c, %	1.9 (1.8, 2.1)	1.9 (1.8, 2.1)	2.0 (1.8, 2.2)	0.912
TG, mmoL/L	1.2 (0.9, 1.8)	1.1 (0.8, 1.7)	1.2 (0.9, 1.8)	0.877
TC, mmoL/L	4.6 (3.9, 5.2)	4.6 (3.7, 5.0)	4.5 (3.9, 5.1)	0.793
LDL-C, mmoL/L	2.8 (2.4, 3.3)	2.9 (2.2, 3.4)	2.8 (2.3, 3.2)	0.855
HDL-C, mmoL/L	1.1 (1.0, 1.3)	1.2 (1.0, 1.5)	1.0 (0.9, 1.2)	0.763
Audiogram type, *n* (%)				0.136
Ascending	97 (18.7)	47 (20.8)	70 (23.8)	
Descending	87 (16.7)	26 (11.5)	41 (13.9)	
Flat	126 (24.2)	66 (29.2)	60 (20.4)	
Profound	210 (40.4)	87 (38.5)	123 (41.8)	

BMI, Body Mass Index; SBP, Systolic Blood Pressure; DBP, Diastolic Blood Pressure; FBG, Fasting Blood Glucose; TG, Triglyceride; TC, Total Cholesterol; LDL-C, Low-Density Lipoprotein Cholesterol; HDL-C, High-Density Lipoprotein Cholesterol.

### Association between uric acid and hearing outcomes

**Figure 3:**
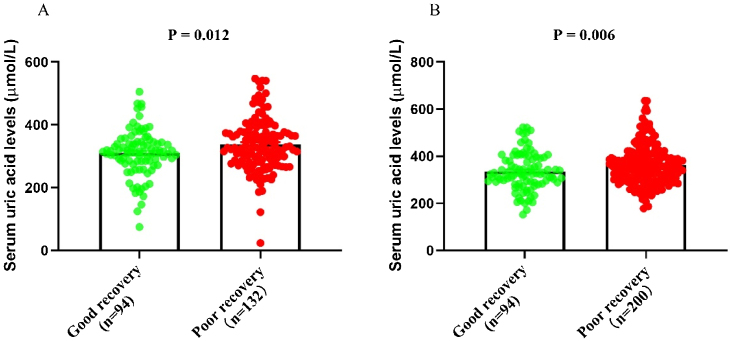
The serum uric acid levels in good recovery and poor recovery groups in female (A) and male (B) patients with SSNHL. SSNHL, Sudden Sensorineural Hearing Loss.

According to the serum uric acid levels, 186 female SSNHL patients were included in the normoglycemia group, and 40 females were divided into the hyperuricemia group. As Table 2 presented, the uric acid levels, BMI, rate of hyperlipemia, and TC were higher in the hyperuricemia group than normouricemia group (*p* < 0.05). In male patients, 237 subjects were categorized into the normouricemia group, and 57 patients were included in the hyperuricemia group. The uric acid levels were markedly increased in the hyperuricemia group than normouricemia group (*p* < 0.05). After audiological following-up, 50, 37, 56, and 43 females in the normouricemia, and 0, 7, 23, and 10 females in the hyperuricemia group obtained complete recovery, partial recovery, slight recovery, and no recovery, respectively. According to Table 3, the rate of good recovery was significantly lower in the hyperuricemia group than the normouricemia group, while the rate of poor recovery and slight recovery was higher in the hyperuricemia group than the normouricemia group both in females and males (*p* <  0.05). Moreover, Figure 2, Figure 3 indicated that serum uric acid levels were significantly different in the four recovery subgroups in female and male patients, and serum uric acid levels were markedly enhanced in the poor recovery group than good recovery group in both genders (*p* <  0.05).

**Table 2:** Demographics and clinical characteristics between normouricemia and hyperuricemia groups.

	Female			Male		
Variables	Normouricemia (*n* = 186)	Hyperuricemia (*n* = 40)	*p*-value	Normouricemia (*n* = 237)	Hyperuricemia (*n* = 57)	*p*-value
Uric acid, μmoL/L	310.7 (270.0, 339.0)	438.1 (404.9, 476.9)	<0.0001	325.6 (289.0, 366.7)	467.2 (442.4, 510.1)	<0.001
Age, year	45.3 ± 12.8	45.0 ± 11.0	0.976	45.0 (35.0, 51.0)	46.0 (41.0, 51.0)	0.758
BMI, kg/m^2^	23.2 (21.6, 25.3)	23.7 (21.1, 25.6)	0.044	22.4 (21.2, 25.1)	22.8 (21,2, 23.1)	0.208
Onset of treatment, day	11.5 (5.0, 14.0)	7.0 (3.5, 12.0)	0.053	8.1 ± 5.3	6.9 ± 5.2	0.119
Side, left/right	97/89	25/15	0.294	115/122	27/30	0.884
Initial hearing threshold, dB	54.8(36.2, 76.9)	55.7 (38.7, 87.2)	0.762	56.8 (38.9, 82.3)	54.8 (36.9, 82.5)	0.874
Contralateral hearing threshold, dB	20.5 (15.2, 25.0)	21.8 (16.4, 28.3)	0.143	20.1 (15.6, 25.4)	21.3 (15.9, 27.2)	0.287
Tinnitus, *n* (%)	122 (65.6)	27 (67.5)	0.856	114 (60.8)	42 (73.7)	0.092
Vertigo, *n* (%)	64 (34.4)	14 (35.0)	1.000	82 (34.6)	18 (31.6)	0.756
Diabetes mellitus, *n* (%)	24 (12.9)	5 (12.5)	1.000	32 (12.5)	5 (8.8)	0.384
Hypertension, *n* (%)	35 (18.8)	5 (12.5)	0.493	39 (16.5)	15 (26.3)	0.089
Hyperlipemia, *n* (%)	36 (19.5)	16 (40)	0.007	48 (20.3)	14 (24.6)	0.473
Creatinine, μmoL/L	62.6 (56.0, 70.0)	65.0 (58.0, 78.0)	0.098	80.2 (71.0, 87.0)	83.0 (75.0, 93.0)	0.116
Urea, mmoL/L	4.4 (3.8, 5.3)	4.6 (3.9, 5.7)	0.108	4.7 (4.0, 5.8)	5.1 (4.2, 6.1)	0.056
SBP, mmHg	122.0 (114.0,134.0)	122.5 (110.0, 131.5)	0.454	122.0 (112.0, 133.0)	127.1 ± 17.5	0.200
DBP, mmHg	80.0 (72.0, 85.0)	78.5 (71, 82.5)	0.402	79.0 (73.0, 87.0)	80.4 ± 11.2	0.522
FBG, mmoL/L	4.9 (4.6, 5.3)	5.0 (4.5, 5.4)	0.956	4.9 (4.6,5.4)	4.8 (4.5,5.3)	0.219
HbA1c, %	1.9 (1.8, 2.0)	2.0 (1.8, 2.1)	0.370	2.0 (1.8,2.1)	1.9 ± 0.2	0.274
TG, mmoL/L	1.1 (0.9, 1.7)	1.0 (0.8, 2.0)	0.949	1.2 (0.9, 1.8)	1.3 (1.0, 1.8)	0.430
TC, mmoL/L	4.7 ± 1.0	5.2 ± 1.2	0.017	4.5 (3.9, 5.1)	4.7 ± 1.1	0.532
LDL-C, mmoL/L	2.9 ± 0.7	3.0 ± 0.8	0.190	2.8 (2.3, 3.2)	2.9 (2.4, 3.4)	0.651
HDL-C, mmoL/L	1.1 (1.0, 1.3)	1.1 (0.9, 1.3)	0.817	1.1 (1.0, 1.3)	1.1 ± 0.3	0.313
Audiogram type, *n* (%)			0.505			0.946
Ascending	42 (22.6)	5 (12.5)		55 (23.2)	15 (26.3)	
Descending	20 (10.8)	6 (15.0)		34 (14.3)	7 (12.3)	
Flat	54 (29.0)	12 (30.0)		49 (20.7)	11 (19.3)	
Profound	70 (37.6)	17 (42.5)		99 (41.8)	24 (42.1)	

BMI, Body Mass Index; SBP, Systolic Blood Pressure; DBP, Diastolic Blood Pressure; FBG, Fasting Blood Glucose; TG, Triglyceride; TC, Total Cholesterol; LDL-C, Low-Density Lipoprotein Cholesterol; HDL-C, High-Density Lipoprotein Cholesterol.

**Table 3:** Hearing recovery between normouricemia and hyperuricemia groups, *n* (%).

	Female			Male		
Variables	Normouricemiia (*n* = 186)	Hyperuricemia (*n* = 40)	*p*-value	Normouricemia (*n* = 237)	Hyperuricemia (*n* = 57)	*p*-value
Good response	87 (46.8)	7 (17.5)	0.001	84 (35.4)	10 (17.5)	0.011
Complete recovery	50 (26.9)	0 (0.0)	<0.001	26 (11.0)	2 (3.5)	0.128
Partial recovery	37 (19.9)	7 (17.5)	0.829	58 (24.5)	8 (21.1)	0.111
Poor response	99 (53.2)	33 (82.50	0.001	153 (64.6)	47 (82.5)	0.011
Slight recovery	56 (30.1)	23 (57.5)	0.002	82 (34.6)	29 (50.9)	0.032
No recovery	43 (23.1)	10 (25.0)	0.838	71 (30.0)	18 (31.6)	0.873

### Multivariate analysis for prognostic factors in SSNHL patients

To explore the prognostic factors in SSNHL patients, both female and male patients were further divided into good recovery and poor recovery groups based on hearing outcomes. In female patients, the serum uric acid levels, initial hearing threshold, rate of hyperuricemia, TC, LDL-C, and audiogram type were significantly different between the good recovery and poor recovery groups (*p* <  0.05). In male patients, the uric acid levels, initial hearing threshold, the rate of hyperuricemia, and TC were lower in the good recovery group than in the poor recovery group (*p* <  0.05) (Table 4). The significantly different variables and potentially prognostic comorbidities were further included in the binary logistic regression analysis. The results showed that uric acid levels, initial hearing threshold, and the rate of hyperuricemia were associated with hearing recovery in female and male patients (*p* <  0.05) (Table 5).

**Table 4:** Factors associated with the recovery of SSNHL of each sex group.

	Female			Male		
Variables	Good recovery (*n* = 94)	Poor recovery (*n* = 132)	*p-*value	Good recovery (*n* = 94)	Poor recovery (*n* = 200)	*p*-value
Uric acid, μmoL/L	313.9 (283.5, 345.0)	327.5 (281.9, 377.3)	0.023	324.5 (289, 396.1)	355.1 (301.2, 404.1)	0.028
Age, year	46.2 (39.1, 54.2)	47.0 (41.0, 53.5)	0.193	45.5 (30.0, 54.0)	45.0 (38.0, 50.5)	0.548
BMI, kg/m^2^	23.4 (21.4, 25.2)	23.4 (21.7, 25.3)	0.601	21.8 (21.2, 25.1)	22.5 (21.2, 24.2)	0.943
Onset of treatment, day	9 (4, 13)	12 (5, 14)	0.041	7 (3, 10)	8 (3, 12)	0.115
Side, left/right	57/37	65/67	0.105	50/44	92/108	0.262
Initial hearing threshold, dB	50.8 (35.7, 76.9)	58.0 (39.2, 83.2)	0.005	52.0 (40.2, 82.3)	57.6 (39.5, 82.3)	0.013
Contralateral hearing threshold, dB	21.1 (16.5, 26.3)	20.9 (16.4, 25.7)	0.953	20.0 (15.6, 25.0)	20.5 (16.2, 25.9)	0.249
Tinnitus, *n* (%)	59 (62.8)	90 (68.2)	0.477	59 (62.8)	127 (63.5)	0.898
Vertigo, *n* (%)	27 (28.7)	51 (38.6)	0.156	34 (36.2)	66 (33.0)	0.600
Diabetes mellitus, *n* (%)	11 (11.7)	18 (13.6)	0.693	10 (10.6)	27 (13.5)	0.574
Hypertension, *n* (%)	19 (20.2)	21 (15.9)	0.480	15 (16.0)	39 (19.5)	0.521
Hyperlipemia, *n* (%)	20 (21.5)	32 (24.2)	0.748	17 (18.1)	45 (22.5)	0.445
Hyperuricemia, *n* (%)	7 (7.4)	33 (25.0)	0.001	10 (10.6)	47 (23.5)	0.011
Creatinine, μmoL/L	62.8 (55.0, 69.0)	63.2 (58.0, 73.0)	0.475	80.3 (71.0, 90.0)	62.0 (74.0, 92.0)	0.204
Urea, mmoL/L	4.4 (3.9, 5.4)	4.6 (4.0, 5.5)	0.713	4.8 (4.0, 5.8)	5.0 (4.2, 6.0)	0.643
SBP, mmHg	121.5 (113.0, 137.0)	123.0 (114.0, 132.0)	0.706	124.0 (112.0, 132.0)	122.5 (112.5, 135.0)	0.878
DBP, mmHg	79.5 (71.0, 88.0)	79.0 (73.0, 84.0)	0.723	80.0 ± 10.0	79.9 ± 11.3	0.919
FBG, mmoL/L	4.9 (4.6, 5.2)	5.0 (4.6, 5.4)	0.520	4.9 (4.5, 5.3)	4.9 (4.6, 5.4)	0.466
HbA1c, %	1.9 (1.8, 2.1)	1.9 (1.8, 2.1)	0.370	2.0 (1.8, 2.1)	2.0 (1.8, 2.1)	0.659
TG, mmoL/L	1.1 (0.8, 1.7)	1.2 (0.9, 1.7)	0.246	1.3 (0.9, 1.8)	1.6 ± 1.6	0.883
TC, mmoL/L	4.3 ± 1.0	4.8 ± 1.1	0.016	4.2 (3.9, 5.3)	4.7 (4.1, 5.4)	0.027
LDL-C, mmoL/L	2.8 ± 0.7	3.0 ± 0.7	0.080	2.8 (2.4, 3.3)	2.8 ± 0.7	0.765
HDL-C, mmoL/L	1.1 ± 0.3	1.1 (1.0, 1.3)	0.402	1.2 (1.0, 1.4)	1.2 ± 0.3	0.093
Audiogram type, *n* (%)			0.004			0.369
Ascending	30 (31.9)	17 (12.9)		21(22.3)	29 (14.5)	
Descending	8 (8.5)	18 (13.6)		17 (18.1)	44 (22.0)	
Flat	21 (37.2)	45 (34.1)		17 (18.1)	43 (21.5)	
Profound	35 (37.2)	52 (39.4)		39 (41.5)	84 (42.0)	

BMI, Body Mass Index; SBP, Systolic Blood Pressure; DBP, Diastolic Blood Pressure; FBG, Fasting Blood Glucose; TG, Triglyceride; TC, Total Cholesterol; LDL-C, Low-Density Lipoprotein Cholesterol; HDL-C, High-Density Lipoprotein Cholesterol.

**Table 5:** Potential factors associated with hearing recovery in SSNHL by binary logistic regression.

Variables	Female			Male		
	OR	95%CI	*p*-value	OR	95%CI	*p*-value
Uric acid, μmol/L	2.563	(1.321−6.872)	0.002	3.892	(1.426−8.563)	<0.001
Initial hearing threshold, dB	1.872	(1.112−3.123)	0.027	1.933	(1.099−2.878)	0.033
Diabetes mellitus, *n* (%)	1.765	(0.973−2.913)	0.652	1.5423	(0.785−2.234)	0.865
Hypertension, *n* (%)	1.323	(0.767−2.032)	0.453	1.238	(0.698−2.321)	0.663
Hyperlipemia, *n* (%)	2.302	(0.903−4.982)	0.129	2.652	(0.876−5.872)	0.202
Hyperuricemia, *n* (%)	2.983	(1.521−5.987)	0.006	2.073	(1.483−5.067)	0.002
TC, mmol/L	1.320	(0.892−2.761)	0.097	1.789	(0.982−2.976)	0.404

SSNHL, sudden sensorineural hearing loss; OR, odd rate; CI, TC, total cholesterol.

## Discussion

In this retrospective study, we aimed to explore the association between serum uric acid levels and hearing outcomes in male and female patients with SSNHL. We observed no significant difference in serum uric acid levels among four audiogram types and demonstrated that serum uric acid concentrations were enhanced in SSNHL patients with poor recovery than those with good recovery. Moreover, both female and male patients in the hyperuricemia group obtained poorer hearing prognosis than the normouricemia group, and binary logistic regression results demonstrated that serum uric acid level, initial hearing threshold, and hyperuricemia were independent risk factors for hearing recovery in SSNHL.

The etiology and pathophysiological mechanism of SSNHL have not been fully elucidated, and emerging evidence showed that cardiovascular risk factors were closely involved in its occurrence and prognosis.^4^, ^9^, ^29^ Prior publications reported that elevated uric acid levels and hyperuricemia crucial in the underlying mechanisms of numerous chronic diseases, especially in cardiovascular diseases.^23^, ^30^, ^31^ Recently, a growing number of evidence suggested that uric acid levels and hyperuricemia negatively affected the sensory capabilities and increased the risk of sensorineural hearing loss.^18^, ^19^ Fasano et al.^32^ analyzed the data of laboratory examinations and found that serum uric acid levels were increased in SSNHL patients compared to healthy controls, which suggested uric acid metabolic imbalance was involved in the pathophysiological mechanism of SSNHL. In the present study, we identified that serum uric acid levels were independently associated with hearing outcomes in SSNHL patients, and hyperuricemia was a strong prognostic factor. Concerning a difference that exists between women and men for serum uric acid, we conducted a subgroup analysis based on gender and yielded a more reliable conclusion. Uric acid was a metabolic end product of purines in the human body, its metabolic disturbance was proven to be closely associated with vascular endothelial function and oxidative stress injury.^33^ Previous in vitro and in vivo experiments demonstrated that elevated uric acid might aggravate oxidative stress response, immunosenescence, and inflammation in inner hair cells, and induced cochlear degeneration and sensory disability.^20^, ^34^ Besides, patients with hyperuricemia were proven to be more susceptible to platelet dysfunction, thrombosis, endothelial dysfunction, and vascular dysregulation, suggesting that hyperuricemia might increase the risk of acute vascular events, and be linked with a poor prognosis.^35^, ^36^, ^37^ Therefore, we have reasons to believe that elevated uric acid and hyperuricemia interfere the hearing recovery, but the underlying mechanisms need to be further discovered.

Exploring prognostic factors and developing individualized treatment were research focuses in SSNHL patients. Although previous publications identified many potential factors and biomarkers associated with hearing recovery in SSNHL patients, including blood glucose, blood pressure, and lipids, no consensus was achieved worldwide.^9^, ^38^, ^39^ Actually, the blood glucose, blood pressure, and lipids levels exist marked gender differences, and sex adjustment and subgroup analysis should be constructed when evaluating their prognostic values of these factors.^40^, ^41^, ^42^ In the present study, we conducted all data analysis based on different genders and found that uric acid level, hyperuricemia, and initial hearing threshold were potential prognostic factors in SSNHL. As previous studies described, the initial hearing threshold was verified to be an essential variable linked with hearing prognosis in SSNHL, patients with higher initial hearing thresholds were more likely to get poor therapeutic effects.^26^, ^27^ It was reported that inner hair cell injury was more extensive and serious in SSNHL patients with higher initial hearing thresholds.^5^, ^43^ Besides, most severe and profound SSNHL were believed to be caused by severe microangiopathy or acute vascular events.^44^, ^45^ Thus, these patients had more difficulties obtaining significant structural and functional recovery despite standardized treatment.

There are several limitations to this study. Firstly, it is a retrospective study conducted at a single center, which introduces the possibility of selection bias. Secondly, the specific duration of hyperuricemia and other comorbidities, and the corresponding treatment protocol and treatment duration is incomplete was not determined, which might affect the reliability of the results. Additionally, the study did not account for potential confounding factors that could influence the outcomes. These limitations collectively reduce the overall reliability of the conclusions.

## Conclusion

To our knowledge, the current study was the first one with a large sample size to demonstrate that hyperuricemia might be an independent risk factor for hearing recovery in SSNHL patients. We also found that serum uric acid and initial hearing threshold were negatively associated with the hearing outcome in males and females with SSNHL. Hyperuricemia had the potential to become a new indicator in the prognostic evaluation and risk stratification of SSNHL patients. Further studies are needed to support our conclusion.

## Authors’ contributions

Yandan Zhou contributed to the conception and design of the study. Jie Wen, Zhongchun Yang, Ruifang Zeng and Wei Gong collected the clinical data and analyzed the data. Qiancheng Jing analyzed the results and drafted and corrected the manuscript. All authors contributed to the article and approved the submitted version.

## Funding

This work was supported by the Natural Science Foundation of Hunan Province (2021JJ3075), Hunan Provincial Health Commission Scientific Research Project (202107010164), Research Project of Changsha Central Hospital (YNKY202003), and Scientific Research Fund of Aier Eye Hospital Group (AF2001D13).

## Conflicts of interest

The authors declare no conflicts of interest.
